# Persistence of lipoatrophy after a four-year long interruption of antiretroviral therapy for HIV1 infection: case report

**DOI:** 10.1186/1471-2334-5-80

**Published:** 2005-10-03

**Authors:** Giustino Parruti, Giuseppe Marani Toro

**Affiliations:** 1 Unit of Infectious Diseases, Ospedale Civile "Spirito Santo", Via Fonte Romana 8, 65126 Pescara, Italy

## Abstract

**Background:**

HIV-infected patients on long-term highly active antiretroviral therapy often present peculiar patterns of fat redistribution, referred to as lipodystrophy. In spite of recent investigations, it is not known whether and to what extent the main features of lipodystrophy – that is lipoatrophy of peripheral fat at face, limbs and buttocks, as well as fat accumulation at breasts, abdomen and the dorso-cervical region – can be reversible once clinically manifest.

**Case presentation:**

A 35 year old Caucasian HIV infected female developed severe diffuse lipodystrophy while on highly active antiretroviral therapy. A remarkable increase of breast size, fat accumulation at waist, and a fat pad on her lumbar spine were paralleled by progressive and disfiguring lipoatrophy of face, limbs and buttocks. The patient decided to interrupt her therapy after 20 months, with a stably suppressed viremia and a CD4 lymphocyte count >500/μL. She could carry on a safe treatment interruption for longer than 4 years. Most sites of fat accumulation switched to nearly normal appearance, whereas lipoatrophy was substantially unchanged at all affected sites.

**Conclusion:**

our observation provides pictorial evidence that lipoatrophy may not be reversible even under ideal circumstances. Therefore, strategies to prevent lipoatrophy should be considered when defining therapeutic regimens for HIV infected patients, especially those at high risk.

## Background

HIV-infected patients on long-term highly active antiretroviral therapy (HAART) often present peculiar patterns of fat redistribution, involving both visceral and peripheral adipose tissue, referred to as lipodystrophy [1,2]. This complex condition was recognized shortly after the introduction of HAART; to date, however, it has been poorly elucidated in terms of pathogenesis [3,4]. Its main features are lipoatrophy of peripheral fat at face, limbs and buttocks, and/or fat accumulation at breasts, abdomen and the dorso-cervical region [3-5].

Complex methods for objective measurements of fat deposits on affected sites involve the use of Dual-energy X-ray absorptiometry, Magnetic Resonance Tomography or CT scans. These methods, however, are costly and not always accessible in routine clinical practice. Moreover, they still lack adequate standardization, so that the diagnosis of lipodystrophy more frequently relies on concordant patient's and physician's evaluation [3-5].

Cross sectional cohort studies indicate that lipodystrophy affects some 50% of patients continually treated with first-generation antiretroviral drugs for at least 18–24 months. Combined or severe abnormalities, however, develop only in 5 to 20% of affected patients [3-6], more frequently in older patients, females, patients with more advanced HIV disease and a longer exposure to antiretroviral drugs, especially stavudine and/or indinavir [6-8]. As to the evolution of body fat abnormalities, evidence has been gathered that once body changes become clinically evident, they generally tend to persist, improving only in a minority of cases [9].

Several strategies have been evaluated in clinical trials, with the aim of controlling such a stigmatizing side-effect. Switching to regimens including Nucleoside Reverse Transcriptase Inhibitors other than stavudine and/or a Non-Nucleoside Reverse Transcriptase Inhibitor instead of a Protease Inhibitor yielded objectively measured, significant increases in subcutaneous fat [10-12]; these, however, were not recorded as significant improvements by the affected patients, even after a long follow-up. At the same time, sites of fat accumulation were generally unmodified in parallel observations [10-12]. Some drugs are presently under evaluation for lipodystrophy, including metformine and rosiglitazone, with inconclusive results as yet [3,5]. Finally, it is not clear whether and to what extent treatment interruptions may be helpful once lipodystrophy is clinically manifest.

Here we report on the evolution of HAART-induced severe combined lipodystrophy in one patient who carried out a very long lasting spell of therapy interruption.

## Case presentation

**Figure 1:**
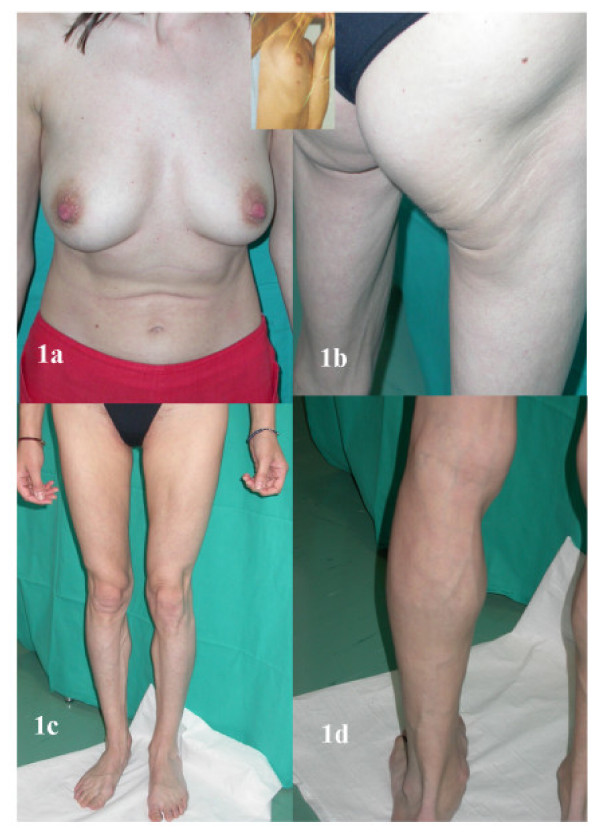
Patient's body habit details at the end of treatment interruption. **1a**. Appearance of breasts. **Insert**. Appearance of patient's breasts before HAART. **1b**. Persistent and marked lipoatrophy at buttocks. **1c**. Lipoatrophy at legs, front view. **1d**. Lipoatrophy of left calf, back view

A 35 year old Caucasian female was found to be infected with HIV1 in 1989, when she tested after heterosexual exposure. She was put on zidovudine in 1995, when her CD4 lymphocyte count steadily declined. She took the drug intermittently until 1997, when she was still asymptomatic, with CD4 lymphocytes <100/μL and a high HIV1 viral load (>500 000 copies/mL). In July 1997, she started her first line HAART regimen including stavudine 30 mg twice a day (bid), lamivudine 150 mg bid and indinavir 800 mg 3 times a day. At that time her body weight was stable, her body shape was lean and preserved, with a body mass index (BMI) of 18.3. Adherence and response to treatment were optimal; she reached undetectable viremia (< 400 copies/mL) and a >200/μL CD4 lymphocyte gain by the 12th week of treatment. Since April 1998, the patient complained about remarkable breast size increase, fat accumulation at waist, and a fat pad grown over her lumbar spine. The pad was 7 × 10 × 3 cm in size by July, 1998. At that time indinavir was replaced with ritonavir/saquinavir 400/400 mg bid due to renal stones. Rapidly progressive, severe lipoatrophy at limbs, buttocks and face ensued in the following months, together with a further increase in size of her fat pads. HIV1 viremia remained constantly undetectable; metabolic parameters were normal throughout follow-up, with an isolated abnormal value of triglycerides (306 mg/dL) in August 1998. Adherence to treatment was full until April 2000, when the CD4 lymphocyte count was >500/μL. The patient was offered a simplified regimen, which she refused, preferring a sharp interruption of HAART. At that time, she did not allow us to take pictures. After interrupting HAART, HIV1 viremia rebounded to lower than baseline, remaining <100 000 copies/mL (mode, 25 000 copies/mL) thereafter. CD4 lymphocyte counts declined very slowly; the patient remained asymptomatic, regularly attending her follow-up visits. Her metabolic parameters, including triglycerides, remained within the normal range at all further checks. She decided to carry on without any treatment for over 4 years, until July 2004, when the CD4 lymphocyte count had dropped to 84/μL. Pads of fat accumulation at waist had reverted to nearly normal appearance; the lumbar pad was no longer appreciable; breasts were still enlarged, although to a lesser extent (Fig. 1a). At variance, lipoatrophy was still well evident; the patient admitted partial improvement at upper extremities, whereas she found no improvement at all for buttocks (Fig. 1b) and legs (Fig. 1c and 1d). Her BMI had dropped to 17.3. The patient was offered a new HAART regimen including nevirapine 200 mg bid, tenofovir diproxil 300 mg once a day and lamivudine 150 mg bid; adherence was full until her last visit, on December 23^th^, 2004, with a CD4 lymphocyte count of 250/μL and a viral load of 1250 copies/mL.

Our observation provides pictorial evidence that severe combined lipodystrophy in our patient was only partially reversible, even under the ideal condition of a long lasting and safe interruption of HAART. Recent reports indicate that the loss of CD4 lymphocytes during treatment interruptions is frequently rapid in patients with a Nadir CD4 lymphocyte count <200/μL and a high rate of cell gain under treatment, irrespective of the level of CD4 lymphocyte count at the interruption of HAART [13,14]. In spite of that, our patient could safely carry her interruption on for over 4 years, remaining asymptomatic throughout the period, with >200 CD4 T-lymphocytes/μL until late in 2003. She did eat a light and balanced diet; she reported taking L-carnitine (5 grams/daily) since June, 2000, until June, 2001, whereas she denied taking any other drug or supplement possibly interfering with lipid metabolism thereafter. Under these extremely favourable circumstances, sites of lumbar and waist fat accumulation reverted to nearly normal appearance, whereas breasts showed partial improvement (Fig. 1a). Such a clear-cut reversal of fat accumulation has not been reported in controlled trials investigating the efficacy of therapy switches [10-12], probably because of persistent interference with lipid metabolism caused by the new antiretroviral drugs [3-5].

Severe lipoatrophy at buttocks and legs, instead, was unchanged both on patient's and on our judgment (Fig. 1b, 1c and 1d). Although it is plausible that instrumental measurements at the affected sites might have detected partial improvements, our observation suggests that the loss of peripheral adipose tissue caused by HAART [3-5,15] may not be fully reversible after treatment interruption, even in the long run.

## Conclusion

Lipodystrophy represents a major drawback of antiretroviral therapy for HIV infection. Its clinical and psychological impact may ultimately jeopardize HAART efficacy in many cases [3,5]. At the time of therapy interruption, our patient declared she would prefer dying rather than living with such disfiguring changes of her body shape. Recent studies systematically addressed the impact of severe lipodystrophy on patients' quality of life, documenting that body habit changes challenge compliance with therapy, as well as social relationships, performance of daily activities, sexuality and self-esteem, especially in young women [16].

As a growing body of clinical evidence suggests that newer HAART regimens may be less toxic on the adipose tissue, causing a clear-cut lower rate of body shape changes in the long run [17], prevention of lipodystrophy should become a key issue when tailoring individual regimens, particularly for patients at high risk of developing fat abnormalities on the basis of the available data.

## List of abbreviations used

μL = microliter

mL = milliliter

bid = bis in die

mg = milligrams

highly active antiretroviral therapy = HAART

## Competing interests

The author(s) declare that they have no competing interests.

## Authors' contributions

GP was in charge of the patient since 1997 at the AIDS Unit in Pescara General Hospital and carried out most of her follow-up visits; report of this case was conceived during a conversation with GMT, who also favoured presentation of the case at the Glasgow meeting in 2004. In depth discussion of pertinent medical literature led to an agreed description of the case and to the draft of manuscript. Both authors read and approved the final manuscript.

## Pre-publication history

The pre-publication history for this paper can be accessed here:


